# Roles for VEGF‐C/NRP–2 axis in regulating renal tubular epithelial cell survival and autophagy during serum deprivation

**DOI:** 10.1002/cbf.3402

**Published:** 2019-06-18

**Authors:** Xiaoyan Chang, Qian Yang, Conghui Zhang, Ying Zhang, Xinjun Liang, Yanyan Liu, Gang Xu

**Affiliations:** ^1^ Department of Nephrology, Division of Internal Medicine, Tongji Hospital, Tongji Medical College Huazhong University of Science and Technology Wuhan China; ^2^ Hubei Cancer Hospital, Tongji Medical College Huazhong University of Science and Technology Wuhan China

**Keywords:** autophagy, NRP–2, serum deprivation, survival, vascular endothelial growth factor C (VEGF‐C)

## Abstract

Vascular endothelial growth factor C (VEGF‐C) is an angiogenic and lymphangiogenic growth factor. Recent research has revealed the role for VEGF‐C in regulating autophagy by interacting with a nontyrosine kinase receptor, neuropilin‐2 (NRP–2). However, whether VEGF‐C participates in regulating cell survival and autophagy in renal proximal tubular cells is unknown. To address this question, we employed a cell modal of serum deprivation to verify the role of VEGF‐C and its receptor NRP–2 in regulating cell survival and autophagy in NRK52E cell lines. The results show that VEGF‐C rescued the loss of cell viability induced by serum deprivation in a concentration‐dependent manner. Furthermore, endogenous VEGF‐C was knocked down in NRK52E cells by using specific small‐interfering RNAs (siRNA), cells were more sensitive to serum deprivation–induced cell death. A similar increase in cell death rate was observed following NRP–2 depletion in serum‐starved NRK52E cells. Autophagy activity in serum‐starved NRK52E cells was confirmed by western blot analysis of microtubule‐associated protein‐1 chain 3 (LC3), immunofluorescence staining of endogenous LC3, and the formation of autophagosomes by electron microscopy. VEGF‐C or NRP–2 depletion further increased LC3 expression induced by serum deprivation, suggesting that VEGF‐C and NRP–2 were involved in controlling autophagy in NRK52E cells. We further performed autophagic flux experiments to identify that VEGF‐C promotes the activation of autophagy in serum‐starved NRK52E cells. Together, these results suggest for the first time that VEGF‐C/NRP–2 axis promotes survival and autophagy in NRK52E cells under serum deprivation condition.

**Significance of the study:**

More researchers had focused on the regulation of autophagy in kidney disease. The effect of VEGF‐C on cell death and autophagy in renal epithelial cells has not been examined. We first identified the VEGF‐C as a regulator of cell survival and autophagy in NRK52E cell lines. And VEGF‐C/NRP–2 may mediate autophagy by regulating the phosphorylation of 4EBP1 and P70S6K. VEGF‐C treatment may be identified as a therapeutic target in renal injury repair due to its capacity to promote tubular cell survival in the future.

**Abbreviations**
4EBP1: 4E binding protein 1
AKI: acute kidney injury
FSGS: focal segmental glomerulosclerosis
LC3: microtubule associated protein 1 light chain 3
NRP–2: neuropilin‐2
p70S6K: ribosomal protein S6 kinase beta‐1
UUO: unilateral ureteral obstruction
VEGF‐C: vascular endothelial growth factor C
VEGFR‐2: vascular endothelial growth factor C receptor‐2

## 1 INTRODUCTION

Tubular cell injury and death are the major lesion of kidney damage caused by ischemia reperfusion injury and nephrotoxicity injury. Proximal tubules located within the outer segment of the out medulla are more susceptible to injury. Investigating mechanisms or new therapeutic molecules underlying the survival of renal tubular epithelial cells is important for the prevention of kidney injury under different stress conditions.

It is noteworthy that renal tubular cells could activate several cytoprotective factors to counteract cellular stress in response to injury. Researches have reported that increased autophagic activity may act as an adaptive self‐protection mechanism of renal tubular cells in response to certain renal injury.^1^, ^2^, ^3^ Autophagy is a cellular process similar to “self‐eating,” which was responsible for the degradation of damaged organelles, long‐lived proteins, and cellular macromolecules through the lysosomal hydrolases. Autophagy plays a vital role in providing nutrients and components for cellular homeostasis and renovation. In kidney, autophagy also coordinates cellular homeostasis and counteracts several types of kidney diseases.^4^, ^5^

Vascular endothelial growth factor C (VEGF‐C), a member of the VEGF family, is the major lymphangiogenic growth factor. The receptor of VEGF‐C includes VEGF receptor‐2 (VEGFR‐2), VEGFR‐3, and neuropilin (NRP–2).^6^ VEGF‐C makes a significant contribution to tumour‐related lymphangiogenesis and lymphatic metastasis in a number of tumour types by mainly acting on VEGFR‐3. Besides lymphangiogenesis, VEGF‐C is also involved in promoting cell survival under stress in many cell types.^7^, ^8^, ^9^, ^10^ A recent research has proved that VEGF‐C protects heart against ischemia‐reperfusion injury via its antiapoptotic effect.^11^ In kidney, VEGF‐C was implicated to ameliorate renal interstitial fibrosis in mouse unilateral ureteral obstruction modal through lymphangiogenesis.^12^

Neuropilins are single‐pass transmembrane, non‐tyrosine kinase receptors, including two homologous members NRP–1 and NRP–2.^13^ As coreceptors for VEGF receptors or Plexins, neuropilins bind different members of the semaphorin family and VEGF family^14^, ^15^ and then play essential roles in axonal guidance, angiogenesis, lymphangiogenesis, and tumour progression.^13^ They are mainly expressed in neuronal tissues, several muscles, kidney, lung, some immune cells, various cancer tissue, and cancer cell lines.^14^, ^16^, ^17^ Studies have confirmed that NRP–2 expression is associated with metastasis and a poor prognosis in a great majority of tumours.^18^, ^19^, ^20^, ^21^ In kidney, increased tubular and interstitial NRP–2 expression was observed in renal biopsy tissues from FSGS patients with various degrees of tubulointerstitial fibrosis.^22^ Upregulation of NRP–2 mRNA in kidney biopsies from patients with nephritic kidney diseases is correlated with worsening renal function and poor renal prognosis.^22^

Notably, VEGF‐C is an important binding ligand for NRP–2.^23^ Studies have indicated that the VEGF‐C/NRP–2 pathway was involved in promoting survival of prostate carcinoma cells under chemotherapy‐induced stress by activating autophagy.^24^ However, the effect of VEGF‐C on cell death and autophagy in renal epithelial cells has not been previously examined. Here, we employed a cell modal of serum deprivation to test the functional role of VEGF‐C and NRP–2 in regulating cell death and autophagy in vitro. Our findings demonstrate that VEGF‐C and NRP2 participate in promoting survival and regulating autophagy in renal epithelial cells under serum deprivation condition.

## 2 RESULT

### 2.1 VEGF‐C protects NRK52E cells against serum deprivation–induced cell death

**Figure 1:**
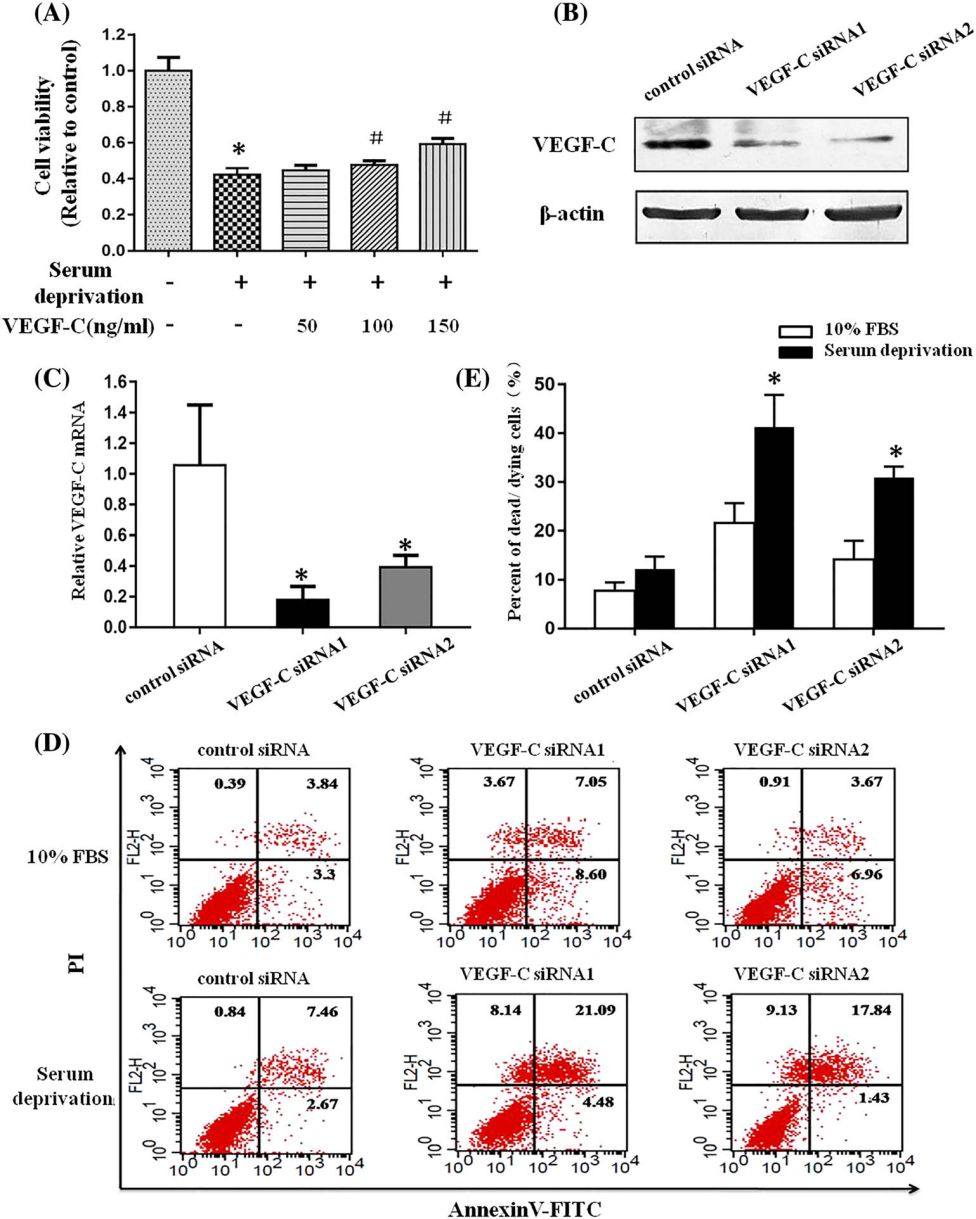
VEGF‐C protects NRK52E cells against serum deprivation–induced cell death. A, VEGF‐C prevented serum deprivation–induced decrease in cell viability. Cells were pretreated with or without different concentrations of VEGF‐C for 4 h and then were cultured in serum (10% FBS) or serum‐free medium with or without VEGF‐C for another 24 h. Cell viability was determined by CCK‐8 assay. All data were presented as the means ± SD of three independent experiments. *P < .05 versus control cells in serum, ^#^ P < .05 versus cells without VEGF‐C treatment in serum‐free medium. B, Western blot analysis of VEGF‐C protein in NRK52E cells after transient transfection with negative control siRNA or VEGF‐C‐specific siRNA oligonucleotides. C, RT‐PCR analysis of VEGF‐C mRNA in NRK52E cells transfected with negative control siRNA or VEGF‐C‐specific siRNA. D, Quantitative assessment of cell death rates by annexin V‐FITC/PI staining. Representative tracings obtained by flow cytometry experiment of three independent experiments of NRK52E cells transfected with negative control siRNA or VEGF‐C‐specific siRNA in serum (10% FBS) or serum‐free medium. E, The results of flow cytometry were shown as quantitative bar graphs. *P < .05 versus negative control group under serum deprivation

We firstly assessed the functional role of VEGF‐C on NRK52E cells under serum deprivation condition by CCK‐8 assay. As shown in Figure 1A, serum withdrawal for 24 hours led to a significant reduction in NRK52E cell viability compared with the cells cultured in medium containing10% FBS. However, the prior addition of recombinant VEGF‐C partly prevented the loss of cell viability induced by serum deprivation in a dose‐dependent manner (Figure 1A). To further prove the prosurvival function of VEGF‐C, we knocked down VEGF‐C expression in NRK52E cells using si‐RNA. Western blot and RT‐PCR were used to confirm the effectiveness of si‐RNA (Figure 1B,C). Next, we explored whether VEGF‐C depletion would lead to increased cell death during serum deprivation by flow cytometry analysis. As shown in Figure 1D and 1E, suppression of VEGF‐C with si‐RNA significantly enhanced cell death rates in serum‐starved NRK52E cells compared with the negative control group. Taken together, these results indicated that VEGF‐C promote NRK52E cells survival under serum deprivation condition.

### 2.2 NRP–2 knockdown enhances cell death in serum‐starved NRK52E cells

**Figure 2:**
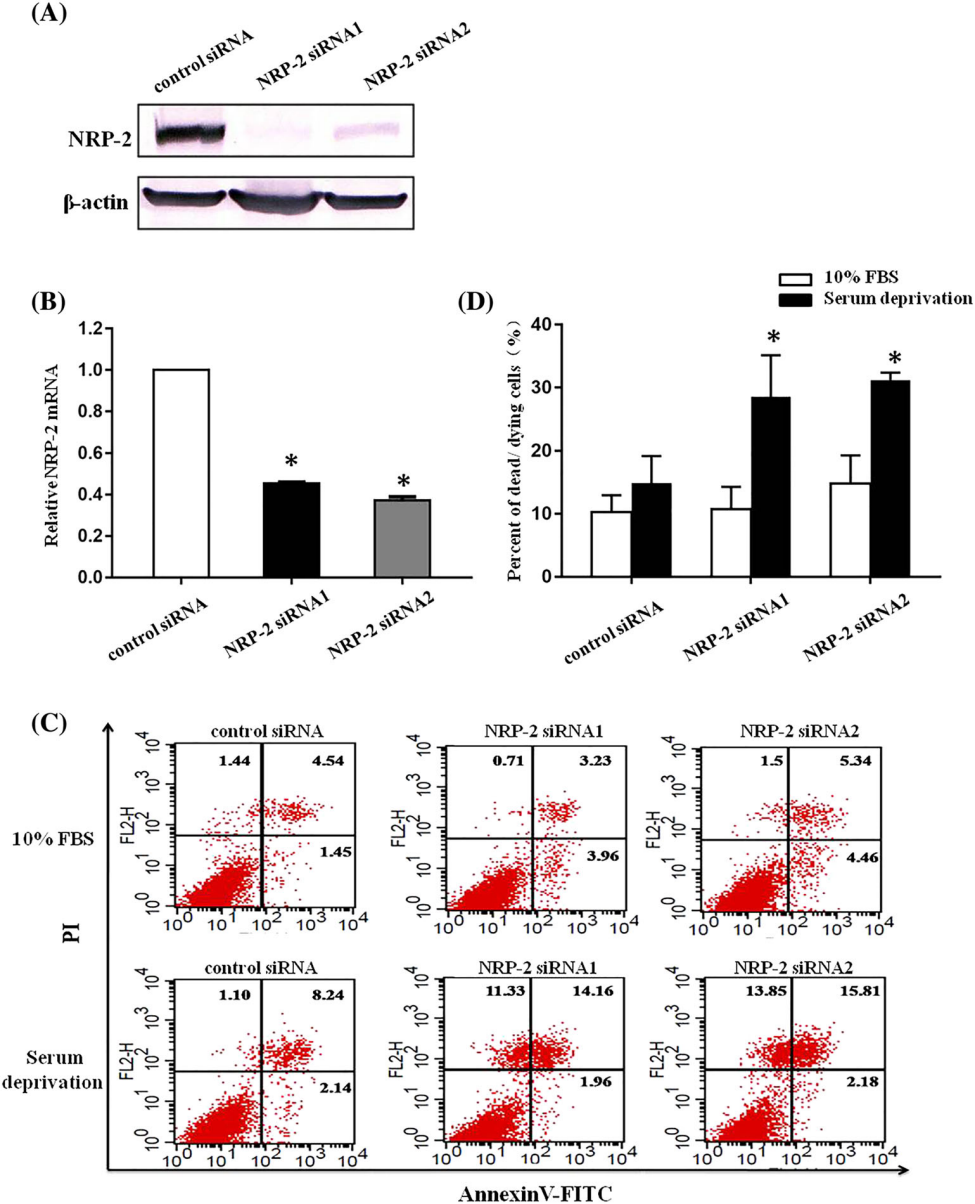
NRP–2 knockdown enhances cell death in serum‐starved NRK52E cells. A, Western blot analysis of NRP–2 protein in NRK52E cells after transient transfection with negative control siRNA or NRP–2‐specific siRNA oligonucleotides. B, RT‐PCR analysis of NRP–2 mRNA in NRK52E cells transfected with negative control siRNA or VEGF‐C‐specific siRNA. C, Quantitative assessment of cell death rates by annexin V‐FITC/PI staining. Representative tracings obtained by flow cytometry experiment of three independent experiments of NRK52E cells transfected with negative control siRNA or NRP–2‐specific siRNA in serum (10% FBS) or serum‐free medium. D, The results of flow cytometry were shown as quantitative bar graphs. *P < .05 versus negative control group under serum deprivation

NRP–2 is expressed on lung cancer, pancreatic cancer, colorectal cancer, prostate cancer cells, and renal tubular epithelial cells.^22^ Previous researches have proved that neuropilin‐2 (NRP–2) plays a critical role in promoting endothelial cell and some types of cancer cell survival.^8^, ^25^, ^26^ We hypothesized that NRP–2 may also participate in regulating the survival of renal tubular epithelial cells. To validate this hypothesis, we examined the effects of NRP–2 knockdown on the viabilities of serum‐starved NRK52E cells. As shown in Figure 2A,B, after specific siRNA transfection, the protein and mRNA levels of NRP–2 in NRK52E cells were obviously decreased. We observed a remarkable decrease in the percentage of viable cells (annexin V–/PI– cells) in the serum‐starved NPR–2 knockdown cells compared with cells transfected with control siRNA (Figure 2C,D). These data suggested that NRP–2 participated in maintaining renal tubular epithelial cell survival during serum deprivation.

### 2.3 Autophagy is induced by serum deprivation in NRK52E cells

**Figure 3:**
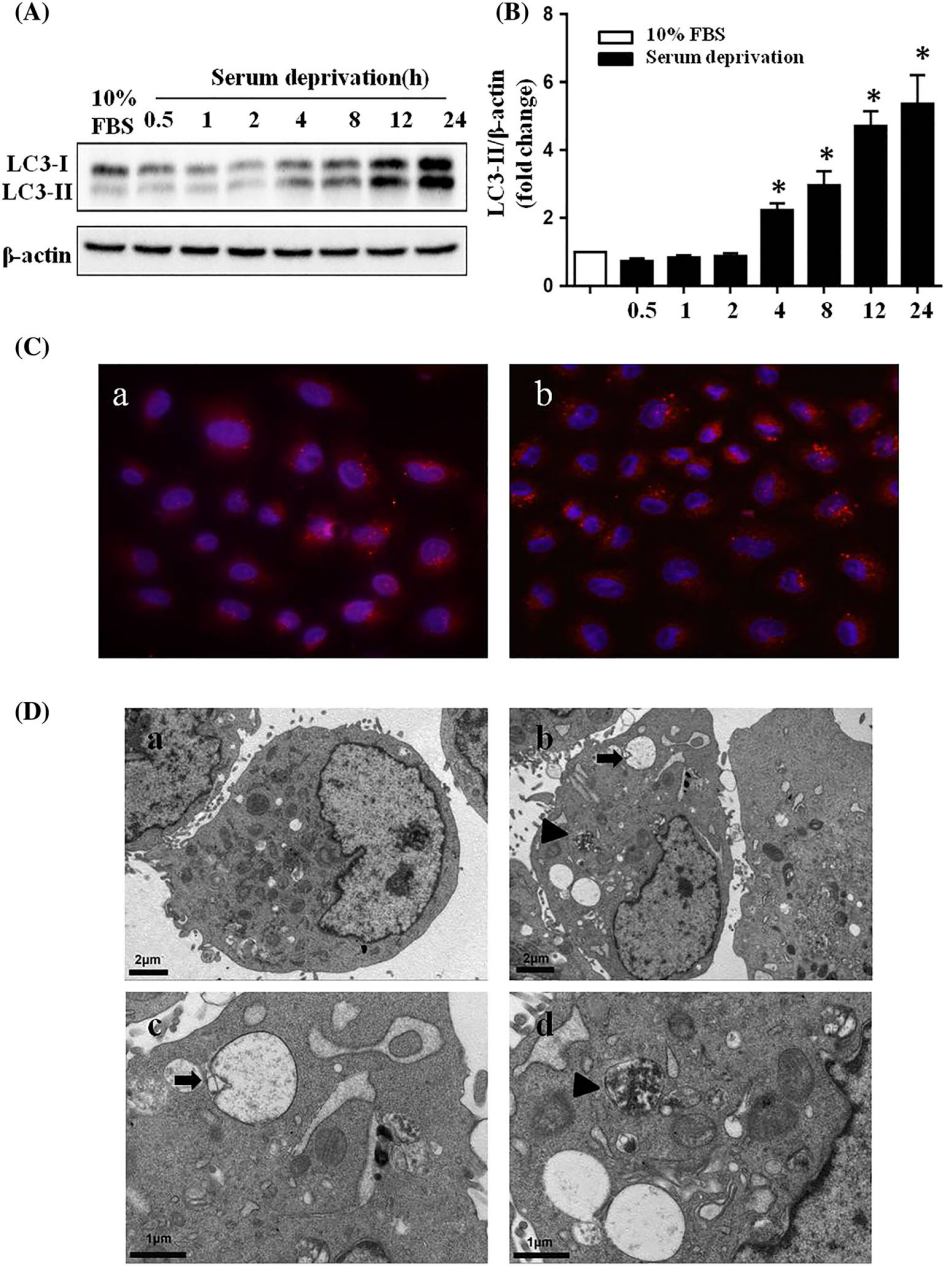
Autophagy is induced by serum deprivation in NRK52E cells. A, Western blot analysis of LC3 expression in NRK52E cells after serum deprivation. NRK52E cells were incubated in serum‐withdrawal medium for various time points to collect the whole‐cell lysates for immunoblot analysis of LC3. β‐actin was used as the protein loading control. B, Quantitation of the optical density of LC3‐II at different time points after serum deprivation. Results are representative of three independent experiments. *P < .05 versus control cells incubated in serum medium. C, Immunofluorescence staining of LC3 in NRK52E cells incubated in serum (a) or serum‐withdrawal (b) medium for 24 h. D, Representative electron micrographs of serum deprivation–induced autophagy in NRK52E cells for 24 h (b‐d). NRK52E cells incubated in 10% FBS medium for 24 h were used as control (a)

Serum deprivation is an efficient inducer of autophagy. The NRK52E cells were incubated in a serum‐free medium for different time periods. We next examined the expression of LC3 by western blot analysis. As shown in Figure 3A,B, incubating NRK52E cells in serum withdrawal media induced a significant LC3 accumulation. Notably, there was a time‐dependent increase in the expression of LC3‐II, which was considered to be the autophagic form of LC3. To further confirm the occurrence of autophagy during serum deprivation, we examined serum‐starved NRK52E cells by fluorescence microscopy and electron microscopy. We used an antibody against endogenous LC3 to detect autophagy in NRK52E cells treated with or without serum deprivation for 24 hours by immunofluorescence. In the control group, LC3 was distributed diffusely throughout the cells, after serum deprivation, notable increase of dot‐like LC3 staining puncta in the cytoplasm was observed (Figure 3C). Additionally, we observed increased autophagic vacuoles by electron microscopy in NRK52E cells after serum deprivation (Figure 3D). Taken together, these data suggested that autophagy was activated by serum deprivation in NRK52E cells.

### 2.4 VEGF‐C and NRP–2 participate in regulating autophagy in NRK52E cells during serum deprivation

**Figure 4:**
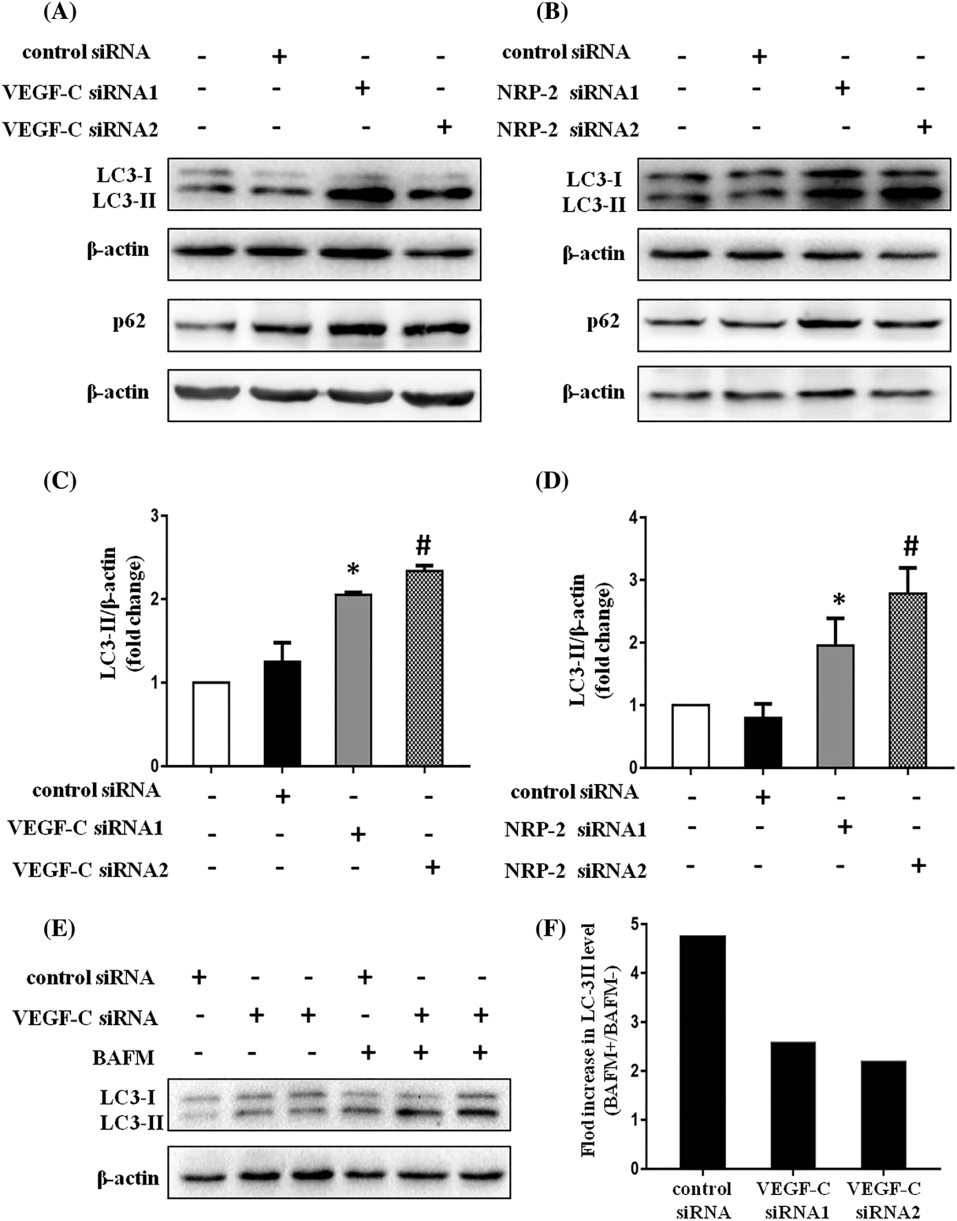
VEGF‐C and NRP–2 participate in regulating autophagy in NRK52E cells during serum deprivation. A, Western blot analysis of LC3 and p62 expression in serum‐starved NRK52E cells which were not transfected, or transiently transfected with negative control siRNA or specific VEGF‐C siRNA. β‐actin was used as the protein loading control. B, Western blot analysis of LC3 and p62 expression in serum‐starved NRK52E cells which were not transfected, or transiently transfected with negative control siRNA or specific NRP–2 siRNA. β‐actin was used as the protein loading control. C, Quantitative assays of A,*P < .05 or ^#^ P < .05 compared with control siRNA group. D, Quantitative assays of B, *P < .05 or ^#^ P < .05 compared with control siRNA group. E, Immmunoblot of autophagic flux analysis in VEGF‐C–depleted NRK52E cells under serum deprivation condition. F, The value of the fold change in LC3‐II in control siRNA transfected NRK52E cells or VEGF‐C depleted NRK52E cells after bafilomycin A1 treatment is illustrated graphically

We further explored whether VEGF‐C or NRP–2 was involved in regulation of renal tubular epithelial cells autophagy during serum deprivation. NRK52E cells were transfected with negative control siRNA or VEGF‐C siRNA and then were incubated in serum‐free medium for 24 hours. We observed a significant increase in LC3‐II expression in the VEGF‐C siRNA group compared with the negative control group (Figure 4A,C). Similar results were observed in the NRP–2 siRNA transfected group (Figure 4B,D). Then, we also examined the expression of p62, an elective autophagy substrate. Similarly, VEGF‐C or NRP–2 depletion resulted in an accumulation of p62 in serum‐starved NRK52E cells (Figure 4A,B).

Increased LC3‐II level does not consequentially represent an enhanced autophagic activity, because autophagy is a dynamic process involving the induction the autophagic vesicle‐associated form LC3‐II and its eventual lysosomal degradation during autophagy. So, the elevated LC3‐II level was also observed when autophagy is inhibited as a result of decreased autolysosomal degradation rather than increased autophagosome formation. We used bafilomycin A1, an autophagy inhibitor that blocks autolysosome‐lysosome fusion to assess autophagic flux or activity. CCK‐8 assay verified that bafilomycin A1 caused a significant decrease in cell viability at concentrations above 10 nM under serum deprivation condition for 24 hours (Figure S1). The optimal bafilomycin A1 concentration (10 nM) was used in the subsequent experiment. As shown in Figure 4E,F, VEGF‐C depletion results in a less fold change in the LC3‐II level compared with control siRNA groups in serum‐starved NRK52E treated with bafilomycin A1, suggesting an inhibition of autophagy by VEGF‐C depletion. Overall, these results indicated that VEGF‐C and NRP–2 participated in regulating autophagy in NRK52E cells during serum deprivation.

### 2.5 Effect of knockdown VEGF‐C and NRP–2 on the expression of mTORC1 signalling pathways in serum‐starved NRK52E cells

**Figure 5:**
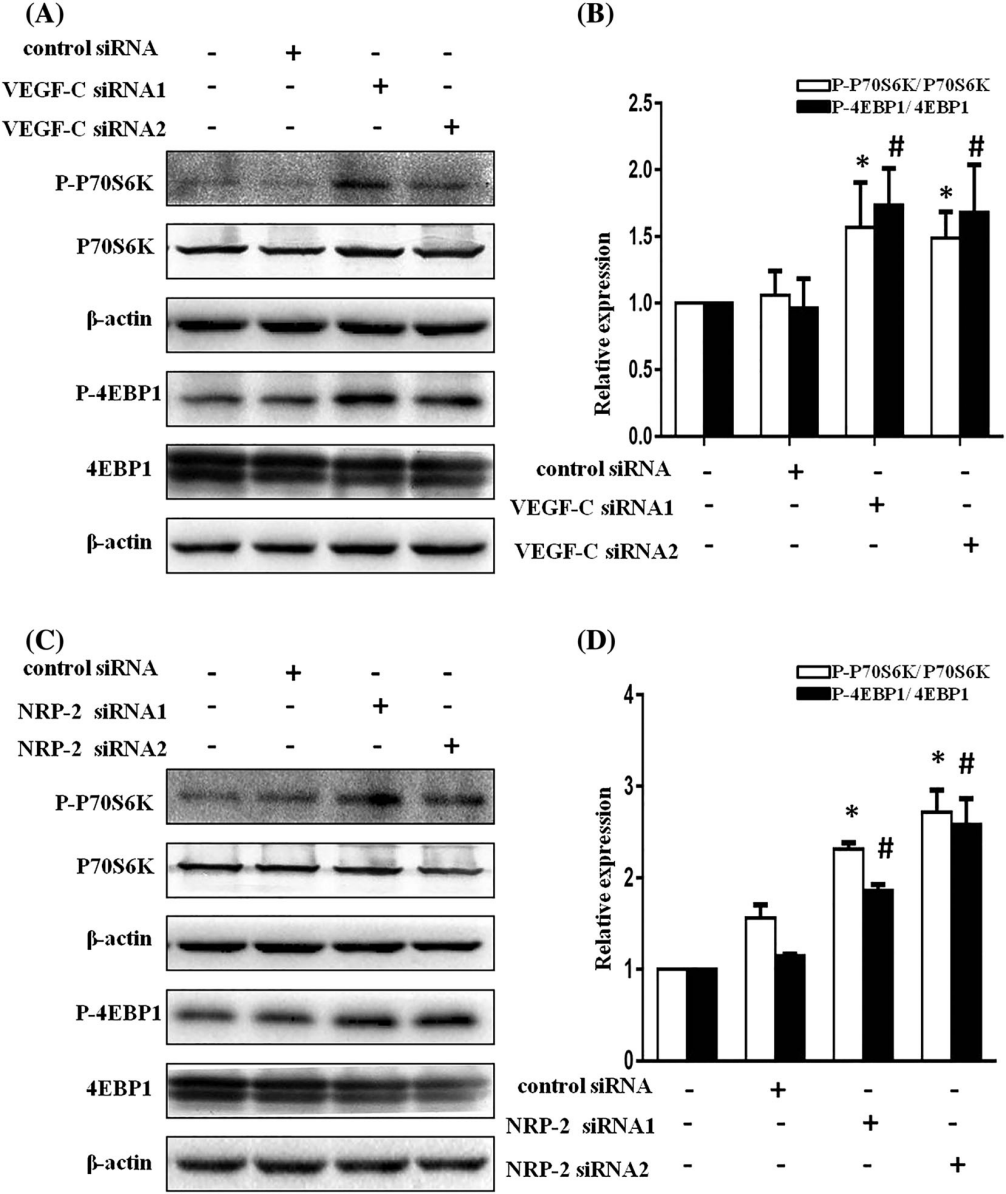
Effect of knockdown VEGF‐C and NRP–2 on the expression of mTORC1 signalling pathways in serum‐starved NRK52E cells. A, Effect of VEGF‐C siRNA on phospho‐P70S6K, phospho‐4EBP1 expression in serum‐starved NRK52E cells was detected with western blot analysis. B, Quantitative assays of A, *P < .05 or ^#^ P < .05 compared with control siRNA group. C, Effect of NRP–2 siRNA on phospho‐P70S6K, phospho‐4EBP1 expression in serum‐starved NRK52E cells was detected with western blot analysis. D, Quantitative assays of C, *P < .05 or ^#^ P < .05 compared with control siRNA group

The Akt/mTOR pathway is essential for the regulation of autophagy. mTOR is evolutionarily highly conserved and forms two different functional protein complexes, mTORC1 and mTORC2. mTORC1 is a key negative regulator of cell autophagy.^27^ So, we examined the effects of VEGF‐C and NRP–2 knockdown on mTORC1 signalling axis by western blot. As shown in Figure 5A,B, an upregulated expression of p‐P70S6K and p‐4EBP1 was observed following the depletion of VEGF‐C in serum‐starved NRK52E cells, indicating an active mTORC1 pathway. The accordant results were observed following the depletion of NRP–2 in serum‐starved NRK52E cells (Figure 5C,D). Thus, these results suggest that VEGF‐C or NRP–2 participate in regulating autophagy by modulating mTORC1 activity.

## 3 MATERIALS AND METHODS

### 3.1 Cell culture and treatment

The rat proximal tubular epithelial cell line, NRK52E, was purchased from Cell Repository, Chinese Academy of Sciences (Shanghai, China). Cells were cultured in Dulbecco's high glucose modified Eagle's medium (HyClone, USA) supplemented with 10% FBS (Gibco, USA), 1% penicillin, and 1% streptomycin. For serum deprivation, when 70% to 80% confluent, NRK52E cells were washed with phosphate buffered saline (PBS) twice and then cultured in serum (10% FBS) or serum‐free medium for indicated periods. Pretreatment of VEGF‐C (Biovision, USA) was carried out 4 hours before serum deprivation, and then NRK52E cells were incubated with VEGF‐C at different concentrations in a serum‐free medium for another 24 hours. For autophagic flux analysis, NRK52E cells transfected with scrambled siRNA or VEGF‐C siRNA were pretreated with 10nmol/L bafilomycin A1 (Sigma‐Aldrich, USA) for 1 hour and then cultured in a serum‐free medium for 24 hours.

### 3.2 Cell viability

Briefly, cell viability was measured with CCK‐8 Counting Kit (DOJINDO Laboratory, Japan). NRK52E cells in logarithmic phase were seeded into 96‐well plates at 1 × 10^4^ cells/well. After treatment according to the protocol, discard the supernatant and wash cells twice with PBS. Ten microliters of the CCK‐8 solution and 100 μL of fresh medium containing 10% FBS were added to each well and incubated at 37°C for 1 to 4 hours. Then, the absorbance at 450 nm was measured by a microplate reader (Bio Teck, USA). Cell viability = [A (treatment) − A (blank)] / [A (no treatment) − A (blank)] × 100.

### 3.3 Western blot analysis

The whole cell extracts were collected by RIPA lysis buffer containing protease inhibitor cocktail. Equal amounts of proteins (30 μg in total) were applied to 12% SDS‐PAGE gel and then transferred to nitrocellulose membranes (Millipore, MA, USA). The membranes were blocked with 5% skim milk dissolved in 0.1% TBS at room temperature for 1 hour and then immunoblotted with different primary antibodies at 4°C overnight: anti‐VEGF‐C diluted at 1:200 (Santa Cruz, CA, USA), anti‐NRP–2 diluted at 1:500 (Abclone,USA). After washing by 0.1%TBS‐T for three times, membranes were incubated with horseradish peroxidase‐conjugated secondary antibodies (1:3000) for 1 hour at room temperature. The proteins were visualized by enhanced chemiluminescence kit. The intensity of bands was analysed with the Image lab software after scanning the bands (Bio‐Rad, USA).

### 3.4 SiRNA for VEGF‐C and NRP–2

The cells were transfected with specific siRNA at 60% to 80% confluence by using Lipofectamine RNAi MAX (Invitrogen, CA, USA). The specific siRNA and negative control siRNA oligonucleotides were purchased from RiboBio (Guangzhou, China). After 48‐hour transfection, cells were harvested for western blot and RT‐PCR analysis. The negative control siRNA was transfected in the same conditions. The sequences of primers are as follows: VEGF‐C‐siRNA1 Sense 5′‐CCAGUGUAGAUG AGCUCAUdTdT‐3′, Anti‐Sense 5′‐dTdTGGUCACAUCUACUCGAGUA‐3′; VEGF‐C‐siRNA2 Sense5′‐GCACAGGUUACCUCAGCAAdTdT‐3′; Anti‐Sense 5′‐dTdTCGUGUCCAAUGGAGUCGUU‐3′; NRP–2‐siRNA1 Sense5′‐GCAAGUUCAA AGUCUCCUAdTdT‐3′, Anti‐Sense 5′‐dTdTCGUUCAAGUUUCAGAGGAU‐3′; NRP–2‐siRNA2 Sense 5′‐GCAUUAUCCUGCCCAGCUAdTdT‐3′, Anti‐Sense 5′‐dTdTCGUAAUAGGACGGGUCGAU‐3′.

### 3.5 RT‐PCR

Total RNA was extracted from NRK52E cells using Trizol (Invitrogen, Life Technologies, USA), and 1‐μg RNA was reverse transcripted into cDNA according to the manufacturer's directions (Promega, Madison, WI, USA). For quantitative PCR, reactions were performed in 25‐μL volumes that contained 12.5 μL of 2× SYBR Green PCR Master Mix (Qiagen, Dusseldorf, Germany). Assays were run on the Roche light 480II Real‐Time PCR Machine. RT‐PCR was carried out with the following primers: rat VEGF‐C, forward 5′‐TCTGGCGTGTTCCTTGCTC‐3′, reverse 5′‐TGCTCCTCCAGGTCTTTGC‐3′; rat NRP–2, forward 5′‐GACGACATTCGGATAAGCACC‐3′, reverse 5′‐GTTCCAATCTCCTTCATAGTCATCA‐3′; rat GAPDH, forward 5′‐GTGGAGTCTACTGGCGTCTTCA‐3′, reverse 5′‐GAGTTGTCATATTTCTCGTGGTTCA‐3′. The relative mRNA expression levels were analysed by the comparative ΔCt method. All experiments were repeated three times (n = 3).

### 3.6 Flow cytometric analysis

After different treatment, cells were collected by pancreatin without EDTA for 1 to 2 minutes. Complete medium was applied to stop the digestion. Then all the medium and cells were harvested into flow tubes, and flow tubes were centrifuged in 2000 rpm for 10 minutes. The supernatant was discarded, and the cells were washed twice by cold PBS. Then 1× binding buffer was utilized to suspend cells and modulate the cell density to 1 × 10^6^/mL; 100‐μL cell suspension was taken to new flow tubes. An annexin V/propidium iodide (PI) apoptosis detection kit (BD Biosciences, CA, USA) was used to quantify the apoptotic and necrotic cell death rate in NRK‐52E cells. Briefly, cells were incubated with 5 μL of annexin V and 1 μL of PI working solution (100 μg/mL) for 15 minutes in the dark at room temperature. Cellular fluorescence was measured by flow cytometry analysis using a flow cytometer (FACSCalibur, BD Biosciences, CA, USA).

### 3.7 Immunofluorescence

NRK52E cells cultured on sterilized glass coverslips in 12‐well plate were incubated overnight at 4°C with indicated primary antibody against LC3 (Abcam, MA, USA; 1:200), followed by incubation with CY3‐conjugated goat antibody (Promoter, Wuhan, China) for 45 minutes at 37°C. Cells were then counterstained with DAPI to visualize the cell nuclei. In the end, the immunofluorescence images were analysed by confocal laser scanning microscopy.

### 3.8 Electronic microscopy

NRK52E cells were fixed with 2.5% glutaraldehyde in 0.1M phosphate buffer overnight at 4°C. The next day, cells were washed three times using 0.1M phosphate buffer, then post‐fixed with 1% osmium tetroxide in 0.1M phosphate buffer (pH 7.4) for 2 hours. The samples were dehydrated through a graded series of ethanol at room temperature, the cells were infiltrated and embedded in spur resin. They were then polymerized with the resin in gelatin capsules at 60°C for 48 hours. Sections of 60–80nm were stained with lead citrate and uranyl acetate for 15 minutes respectively, and then examined using a Hitachi H‐7000FA transmission electron microscope.

### 3.9 Statistical analysis

Results were presented as the mean ± SEM of three independent experiments. Statistical analysis of data was carried out by the *t* test or one‐way analysis of variance (ANOVA) using SPSS (version 18.0). Statistical significance was determined at *P* < .05.

## 4 DISCUSSION

This study was undertaken to illuminate the role of VEGF‐C and its receptor NRP–2 in regulating renal tubular epithelial cell survival and autophagy. VEGF‐C was identified as a key lymphangiogenic factor, mainly acting via VEGF receptor (VEGFR)‐3.^28^ Numerous studies have shown that VEGF‐C promoted tumour metastasis in various malignancies by mediating tumour angiogenesis, lymphangiogenesis, and invasion.^29^, ^30^ In kidney, VEGF‐C participated in lymphangiogenesis in mouse unilateral ureteral obstruction (UUO),^31^ and further study has proved that VEGF‐C could ameliorate renal interstitial fibrosis through lymphangiogenesis in UUO mice.^12^ Additionally, VEGF‐C was involved in mediating chemoresistance in certain types of cancer cells.^9^, ^24^, ^32^ In heart ischemia/reperfusion injury model, VEGF‐C markedly promoted cardiomyocyte survival via the activation of PI3k/Akt signalling pathway.^11^ However, it remains unclear whether VEGF‐C plays a protective role on renal tubular epithelial cells. In the present study, we demonstrated that VEGF‐C inhibits the loss of cell viability induced by serum deprivation in a dose‐dependent manner in NRK52E cells. Besides, the inhibition of VEGF‐C expression using siRNA technology further enhanced cell death rate in NRK52E cells under serum deprivation conditions. These results suggested that VEGF‐C protects tubular epithelial cells from serum deprivation–induced cell death in vitro.

NRP–2, a well‐known receptor for semaphorins, is also an important independent receptor or coreceptor that interacts with vascular endothelial growth factors. NRP–2 exerts vital functions in lymphatic endothelial cells, neurons, and tumour cells. Studies have shown that NRP–2 overexpression was closely correlated with tumour lymphangiogenesis and lymphatic metastasis in different types of cancer cells.^18^, ^19^, ^20^, ^21^, ^33^ So, NRP–2 was considered to be a novel target for cancer therapy. Previous studies have found that NRP–2 could act as a coreceptor that promote survival and migration in human endothelial cells.^25^ Besides, Muders et al proved that VEGF‐C/NRP–2/AKT‐1 axis is involved in protecting prostate cancer cells from H_2_O_2_‐induced oxidative stress.^8^ NRP–1 and NRP–2 are also expressed in human kidneys. However, there is a paucity of data on the functional role of NRP–2 in renal pathophysiology. Schramek et al reported an upregulation of tubular and interstitial NRP2 expression in human focal segmental glomerulosclerosis (FSGS) tissues. They also proved that elevated mRNA expression of NRP2 in kidney biopsies correlated with a more severe impaired renal function and a poor renal outcome in several nephrotic kidney diseases.^22^ We have proved that VEGF‐C is crucial for renal tubular survival, so we next wanted to analyse whether NRP–2 participates in regulating cell survival in NRK52E cells. We knocked down NRP–2 expression in NRK52E cells using siRNA and confirmed it by western blot and RT‐PCR. NRP–2 expression was obviously attenuated in cells transfected with NRP–2 target siRNA. The cell death rate after NRP–2 knockdown was quantitated using flow cytometric analysis. Similarly, the cell death rate was significantly increased in the serum‐starved NRP–2 siRNA group compared with the serum‐starved negative control siRNA group. These data indicated that NRP–2 participate in promoting survival of NRK52E cells under serum deprivation condition.

Autophagy is the “self‐eating” process responsible for the degradation of damaged organelles and cytosolic materials, which helps to maintain cellular homeostasis. Autophagy plays important roles in the physiology and pathogenesis of several kidney diseases, such as ischemia‐reperfusion or nephrotoxins induced acute kidney injury (AKI),^1^, ^2^, ^3^, ^34^, ^35^ diabetic nephropathy,^5^ and glomerular disease.^36^ Previous studies have indicated that VEGF‐C/NRP–2 axis was involved in regulating autophagy and promoting survival of cancer cells under chemotherapy treatment.^24^ However, whether VEGF‐C/NRP–2 participate in regulate autophagy in renal tubular epithelial cells is still unknown. So, we next examined the role of VEGF‐C and NRP–2 in regulating autophagy in NRK52E cells under serum deprivation conditions. We first examined the expression of LC3, which is a marker of autophagy by western blot. Our data demonstrated that LC3‐II levels were increased in a time‐dependent manner under serum deprivation conditions. Besides, increased punctuate endogenous LC3 staining was observed in serum‐starved NRK52E cells by immunofluorescence. The results were further confirmed by electron microscopy. We can observe the accumulation of autophagic vacuoles in serum‐starved NRK52E cells. Our data demonstrated that autophagy was triggered by serum deprivation in NRK52E cells.

We next explored whether VEGF‐C or NRP–2 was involved in the regulation of autophagy in NRK52E cells. Our study demonstrated that knockdown of VEGF‐C further enhanced serum deprivation–induced LC3‐II expression. A similar increase of LC3‐II expression was observed in serum‐starved NRK52E cells after NRP–2 knockdown. However, LC3‐II accumulation may be caused by increased autophagosome formation or impaired lysosomal degradation. So, we next performed autophagic flux experiments by using baflomycin A1, which prevents downstream autophagosome‐lysosome fusion. We calculated the fold change in the LC3‐II level under serum deprivation condition in the presence or absence of baflomycin A1. After baflomycin A1 treatment, a lower fold‐change in the LC3‐II level in VEGF‐C knockdown group was observed compared with the negative control group, suggesting VEGF‐C depletion inhibited the autophagic degradation, thereby dysregulated autophagy.

mTORC1 was identified as a negative regulator of autophagy by phosphorylating two vital effectors, ribosomal protein S6 kinase beta‐1 (p70S6K) and 4E binding protein 1 (4EBP1).^27^, ^37^ Our results indicated that the levels of p‐4EBP1 and p‐P70S6K proteins were higher in the VEGF‐C depletion groups than in the control siRNA treatment groups. We also found a similar increase in p‐4EBP1 and p‐P70S6K expression following NRP–2 depletion under serum deprivation conditions. Hence, we speculated that VEGF‐C/NRP–2 were involved in autophagy via regulating mTORC1 activity.

In conclusion, our experiment identifies the role of VEGF‐C and NRP–2 as a regulator of cell survival and autophagy in NRK52E cell lines. VEGF‐C treatment may be identified as a therapeutic target due to its capacity to promote renal tubular epithelial cell survival. Furthermore, VEGF‐C/NRP–2 may mediate autophagy by regulating the phosphorylation of 4EBP1 and P70S6K in NRK52E cells. Our research has some limitations. More researchers should be focused on the mechanisms that link the regulation of cell death and autophagy in our experiment. The in vitro model in our experiment may not fully reflect the pathological and physiological changes in vivo. Besides, we did not verify the role of VEGF‐C/NRP–2 in animal models. Furthermore, whether other tyrosine kinase receptors of VEGF‐C were involved in this process is unknown.

## CONFLICT OF INTEREST

The authors declare that the research was conducted in the absence of any commercial or financial relationships that could be construed as a potential conflict of interest.

## FUNDING

This work was partly supported by the National Natural Science Foundation of China (nos. 81372244, 81572287, and 81470948), international (regional) cooperation and exchange projects (NSFC‐DFG, grant no. 81761138041), the major research plan of the National Natural Science Foundation of China (grant no. 91742204), and the National Key Research and Development Program (grant no. 2016YFC0906103).

## Supporting information

Figure S1. The effect of Baflomycin A1 on NRK52E cell viability under serum deprivation condition. Line graph showed the viability of serum‐starved NRK52E cells stimulated with different concentrations of bafilomycin A1 for 12 or 24 h. Values are means ± SE. *P < .05 or * * P < .01 versus control cells incubated in serum medium.
