# Supplementary material for: Roles for VEGF‐C/NRP–2 axis in regulating renal tubular epithelial cell survival and autophagy during serum deprivation
Source: Cell Biochem Funct. 2019 Jun 18;37(4):290–300. doi: 10.1002/cbf.3402 (PMC6618243; doi:10.1002/cbf.3402)
Supplement: Supplementary file 1 — Figure S1. The effect of Baflomycin A1 on NRK52E cell viability under serum deprivation condition. Line graph showed the viability of serum‐starved NRK52E cells stimulated with different concentrations of bafilomycin A1 for 12 or 24 h. Values are means ± SE. *P < .05 or * * P < .01 versus control cells incubated in serum medium. [file CBF-37-290-s001.docx]

**
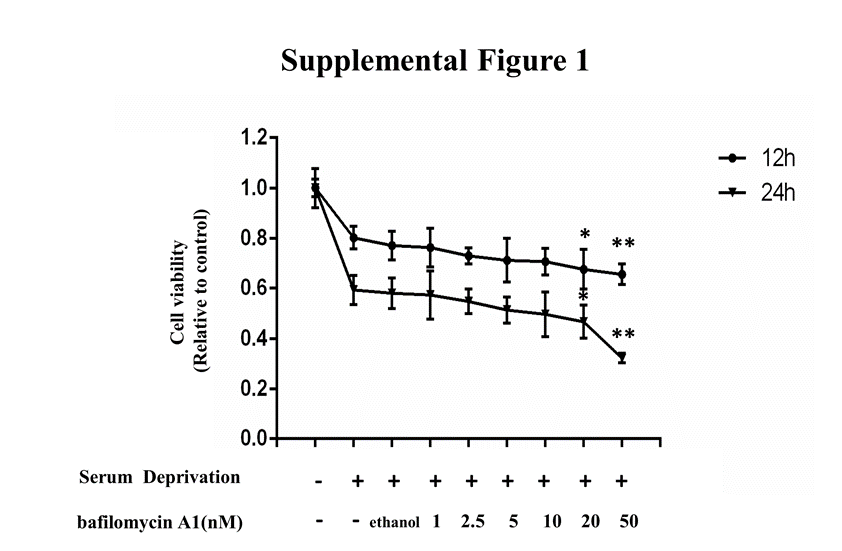
 Supplemental Figure 1. The effect of BAFM on NRK-52E cells viability under serum deprivation condition.** Line graph showed the viability of serum-starved NRK-52E cells stimulated with different concentration of bafilomycin A1 for 12h or 24h. Values are means ± SE. *P<0.05 or * * P<0.01 versus control cells incubated in serum medium.
